# A GI‐Interoceptive Threat Theory of Restrictive Eating: Insights From Active Inference and Chronic Pain

**DOI:** 10.1002/brb3.70892

**Published:** 2025-09-21

**Authors:** Laura Case

**Affiliations:** ^1^ Department of Anesthesiology University of California San Diego San Diego California USA; ^2^ VA San Diego Healthcare System San Diego California USA

**Keywords:** active inference, central sensitization, gastrointestinal, interoception, restrictive eating

## Abstract

**Introduction:**

Restrictive eating is a common eating disorder (ED) behavior and risk factor. Disturbed body image is also highly associated with the development and maintenance of eating disorders. Yet body image often remains distorted after treatment, and there is little scientific understanding of the mechanisms by which restrictive eating and distorted body image are linked. In parallel, current models of chronic pain describe how fear and negative beliefs about pain lead to avoidance of painful sensations and a threatened response to their occurrence, entrenching a cycle of amplified pain that risks becoming chronic. These models are informed by theories of active inference, which describe how the brain actively shapes sensory experience to reduce prediction errors (discrepancy between predictions and sensory data). This understanding has led to significant advances in the treatment of chronic pain.

**Methods:**

Theories of active inference and central sensitization in chronic pain and ED research on fear of fatness and gastrointestinal (GI) interoception were reviewed and integrated to propose a theoretical model of sensitization of GI sensations in EDs.

**Results:**

Through the lens of active inference, I propose a hypothesis‐generating framework that a fatphobic culture confers beliefs that fatness is bad, driving avoidance. Through dieting—an attempt to avoid fatness—interoceptive sensations of fullness/distention come to signify fatness and are thus construed as threatening. Similar to chronic pain, these sensations become amplified and persistent, distorting body image and entrenching restrictive eating patterns. This framework leads to novel proposals for research and treatment.

**Conclusions:**

Significant theoretical advances may be afforded by considering EDs through models of central sensitization and active inference in chronic pain. I propose mechanistic links between interoception, distorted body image, and restrictive eating, and discuss implications and future directions for research and treatment.

## Graphical abstract

In this theory/review paper, I borrow from leading theories of central sensitization in chronic pain and predictive inference in affective neuroscience to propose a theoretical model of interoceptive‐affective sensitization of gastrointestinal sensations in eating disorders.

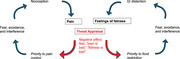

## 1 Introduction

Eating disorders (EDs) affect 1%–3% of women (American Psychiatric Association 2000; Hudson et al. 2007; Keski‐Rahkonen et al. 2009; Qian et al. 2022) and are associated with substantial and costly medical morbidity (McKenzie and Joyce 1992), high rates of comorbid psychopathology, significant psychosocial impairment (American Psychiatric Association 2000; Wonderlich and Mitchell 1997), and the ED anorexia nervosa (AN) carries the highest mortality rate of any psychiatric disorder (Birmingham et al. 2005, Papadopoulos et al. 2009; Sullivan 1995). Restrictive eating is a common ED behavior as well as a fairly transdiagnostic risk factor for EDs (S. B. Wang et al. 2021). Disturbed body image is also highly associated with the development and maintenance of EDs (Stice and Shaw 2002) and predicts long‐term outcome in AN (Boehm et al. 2016). Yet body image commonly remains distorted for individuals in remission from AN (Engel and Keizer 2017). There is no validated therapy for this difficult‐to‐treat symptom, and there is little scientific understanding of the mechanisms by which restrictive eating and distorted body image are linked.

## 2 An Interoceptive Threat Theory of Restrictive Eating

In the current paper, I borrow from current theories of central sensitization (amplification of pain processing in the brain) in chronic pain and active inference (how belief and prediction shape current experience) in affective neuroscience to propose a theoretical model of sensitization of fullness‐related GI sensations in EDs and its implications for ED etiology and treatment. In so doing, I propose mechanistic links between interoception, distorted body image, and restrictive eating. I developed these theories from my background in human neuroscience of pain and affective touch, with additional experience in ED research. As I noticed parallels between amplified perceptions, fear, and avoidance of pain sensations in chronic pain and amplified perceptions, fear, and avoidance of fatness in EDs, I wondered to what extent models of chronic pain could inform ED research and treatment. In preparing the current manuscript, I searched for literature on chronic pain and active inference or central sensitization, as well as on EDs and active inference, central sensitization, interoception, distorted body image, perception of fatness and restrictive eating. The model I present should be considered a conceptual framework for hypothesis generation. It does not seek to supplant existing rigorous, multilevel theoretical accounts of EDs but to complement them by highlighting key somatosensory elements that may improve their explanatory power.

Body image is a multifaceted construct that includes perceptual, affective, and cognitive components. Individuals with AN exhibit both perceptual body distortion (Engel and Keizer 2017) and body dissatisfaction—negative attitudes towards the body (Stice and Shaw 2002). In addition, a “feeling of fatness” explains unique variance in disordered eating behaviors (Linardon et al. 2018). Body image distortion is often conceptualized from a cognitive perspective. However, a “feeling of fatness” is a physically embodied sensation that is rooted in both somatosensory pathways and cognitive interpretation (Mehak and Racine 2020). Although “feeling fat” may be characterized clinically as a misperception of sensations or emotions, I draw upon advances in the neuroscience of chronic pain to propose that these feelings are rooted in gastrointestinal (GI) interoceptive sensations that become amplified and distorted, parallel to central sensitization in chronic pain and its distortion of body image (Moseley 2008). In a fatphobic culture, most individuals seek to avoid fatness to avoid weight‐based discrimination and victimization (Puhl and Suh 2015). I propose that signals of gastric distention—as well as other skin or deep tissue‐related sensations related to fullness or satiation—become interpreted as sensations of fatness, and thus desirable to avoid. Fear and avoidance amplify central processing of sensations linked to “fatness” and attach a negative valence to them. Amplification and fear‐avoidance would then lead to chronic feelings of fatness as well as consequent distortions in body perception and sensory perception that are observed in AN (Keizer et al. 2012) as well as other EDs.

In addition, I draw from theories of active inference and the role of interoception in affect. Active inference stipulates that mental experience is largely predictive in nature; the brain continuously predicts sensations and actions, and this prediction influences perception. The brain actively shapes sensory experience to reduce prediction errors (discrepancies between predictions and actual sensory data). Active inference is exemplified when we errantly think we recognize a friend in a crowd, as our predictions bias us to perceive faces we are familiar with, or when we begin to sweat when we enter a haunted house, expecting that we will be frightened. Active inference minimizes energy expenditure in response to unexpected circumstances and maximizes homeostasis of the brain and body. However, it also means that much of our mental experience is shaped by our beliefs and past experiences. These “priors”—neural encodings of our beliefs and expectations about the world—can be adjusted over time through prediction errors, neural calculations of discrepancy between our prediction and incoming sense data. A hyper‐prior is a strong cognitive belief that shapes downstream priors (Veissière et al. 2020). In Western culture, a highly salient and pervasive hyper‐prior is that certain patterns of eating lead to fatness and that fatness is bad (especially in women). Thus, it is unsurprising that many young people, and especially girls, restrict their eating in an attempt to avoid fatness.

I propose the following model—intended as a hypothesis‐generating framework—to explain the development of persistent feelings of fatness:

1. Fatphobic hyperpriors drive a fear of fatness.
2. Gastric stretch sensations become salient and threatening according to whether they seem to indicate fatness: “good” to hunger or emptiness; “bad” to gastric distention or satiety.
3. Dieting links GI sensations to negative affect, given the positive or negative reinforcement of weight changes. Gastric stretch drives negative affect and avoidance.
4. Gastric stretch sensations become amplified and persistent via central sensitization (parallel to chronic pain), altering body image and body representation.

Below, I elaborate on this model framework, discussing evidence, gaps, and future directions for research and treatment.

### 2.1 Fatphobic Hyperpriors Drive a Fear of Fatness

Sensory perception is constantly predicted and modified by neural circuits that instantiate our expectations, or *priors*. The brain uses probabilistic prediction to model its needs and predict changes, optimizing metabolic expenditure. In each moment, sensory data are compared to prediction, and discrepancies are registered as prediction errors (essentially, surprise). Sensory data and predictions are each weighted by their precision, with higher precision data afforded greater ability to modify future predictions (Papadopoulos et al. 2009; Sullivan 1995; S. B. Wang et al. 2021; Stice and Shaw 2002). Applied to internal body perception (interoception), interoceptive predictions establish homeostatic setpoints against which new interoceptive data are compared, with prediction errors driving autonomic responses. Interoceptive prediction and perception occur in the insula, and its integration with exteroceptive sensory data shapes higher‐level body representation (Keizer et al. 2012; Veissière et al. 2020; Becker and Stice 2017; Jarman et al. 2021; Lin et al. 2023).

Unless actively resisted (Becker and Stice 2017), a culture of prescriptive thinness confers hyperpriors that one's body *should* be thin and that fatness is bad and scary. Internalization of “ideal” appearances online, for example, has been shown to increase body dissatisfaction (Jarman et al. 2021). Similarly, a recent study found that external pressure and an internalized ideal to be thin/fit were common triggers for AN (Lin et al. 2023). Across ED models, both preoccupation with weight and shape (i.e., weight and shape concerns, thin body preoccupation, body dissatisfaction, body image disturbance, appearance anxiety, and body shame) and negative affect are considered two of the most prominent risk factors that contribute to the development of EDs (Pennesi and Wade 2016). These precise hyperpriors naturally lead to a fear of fatness and attempts to avoid fatness. If thinness is compulsory, then interoceptive data will be monitored for violations in the form of fatness, and discrepancies will register as prediction errors, motivating emotional and behavioral responses to perceived fatness.

A striking parallel exists to current models of chronic pain. Pain‐like fatness—is culturally understood as scary and undesirable, and even potentially disabling. Past pain experiences often lead to fear of pain as well as fear of movement that might elicit pain (kinesiophobia). Notably, avoidance of movement can amplify central processing of pain signals and deprive the patient of a chance to update their active inference models of movement and pain. Fear and avoidance of pain consequently predict higher pain intensity (Kroska 2016) and disability in chronic pain (Zale and Ditre 2015). Similar processes of fear and avoidance occur in post‐traumatic stress disorder (PTSD), as sufferers often avoid or dissociate from internal bodily signals after trauma, allowing expectations of pain and trauma to persist rather than be updated by innocuous sensory experiences (Strigo and Simmons 2024).

### 2.2 Gastric Stretch Sensations Become Salient and Threatening

GI sensations are intuitively more relevant to ED symptomatology than other interoceptive functions and have recently been the subject of calls for increased focus on clinical ED treatment (Khalsa et al. 2022). Individuals with AN frequently exhibit altered GI sensation, including exaggerated fullness after small meals, early satiety, and abdominal pain (Schalla and Stengel 2019). This exaggerated fullness decreases after treatment (Salvioli et al. 2013), suggesting a potential biomarker of treatment progress. Feelings of gastric distention/fullness arise from mechanical deformation of vagal nerve terminals in the stomach that respond rapidly to stretch and tension of the stomach wall (reviewed in Gautron 2021). Parallel to pain processing, central processing of gastric distention has been linked to the insular cortex (G.‐J. Wang et al. 2008; Ladabaum et al. 2007; Tataranni et al. 1999). However, while GI interoception has been discussed in the context of active inference, the cause of interoceptive differences in EDs has not been explained (Khalsa et al. 2022).

I propose that a fear of fatness leads vulnerable individuals to assign a threat value to any interoceptive cues believed to signal fatness. Fear and anxiety are commonly directed at the body in EDs—which has been theorized as representing a threatening object for individuals with AN (Osler 2021)—and also at food, food intake, weight gain, or becoming fat (Charland et al. 2013, Espeset et al. 2012; Arnaud et al. 2023). Disgust towards one's body is predictive of body image disturbance (Stasik‐O'Brien and Schmidt 2018) and may motivate behaviors to reduce bodily disgust (Arcelus et al. 2011; Anderson et al. 2021). In addition, changes in GI‐specific—but not general—physical anxiety sensitivity predict both lower ED symptoms and lower trait anxiety at discharge and 6‐month follow‐up (Velkoff et al. 2024). These findings point to the importance of GI‐specific anxiety sensitivity as a potential maintaining factor in EDs.

A recent application of active inference to EDs has proposed that individuals with AN experience noisier interoceptive signals, which lead them to restrict their eating to amplify hunger signals in an effort to minimize interoceptive certainty and the construction of a coherent self via interoception (Barca and Pezzulo 2020). However, this model fails to explain why eating restriction is the strategy pursued to reduce interoceptive confusion or why the feeling of fatness is pervasive and persistent in EDs. Indeed, mistrust in one's interoceptive signals and not feeling safe in one's body are associated with ED symptoms (T. A. Brown et al. 2020). While such mistrust could reflect interoceptive uncertainty, I propose that it reflects a perceived threat signal and negative valence of *specific* GI symptoms associated with fullness/fatness. If thinness is expected and fatness feared, then interoceptive signals associated through conditioning with weight gain will generate prediction errors that feel like fatness. These prediction errors will increase focus on interoceptive sensations.

### 2.3 Dieting Links GI Sensations to Negative Affect, Driving Avoidance

Leading neuroscientific theories of emotion stipulate that emotion is constructed from simulations of physiological needs (Barrett 2017) and thus from interoceptive signals from the body (Craig 2003). Indeed, interoceptive sensations are usually experienced as lower‐dimensional feelings of affect (Barrett 2017; Barrett and Bliss‐Moreau 2009), implying that negative emotions about the physical body are inextricably linked to incoming sensations of the body. This makes it highly plausible that interoceptive feelings of fullness and distention drive negative affect in individuals with weight and shape concerns. In this fatphobic context, fear of fatness would shape the emotional implications of hunger and fullness. Signals of fullness—interpreted as relating to weight gain—would increasingly evoke negative emotions such as fear, disgust, and shame. If this hypothesis is correct, then feelings of fullness should evoke negative affect and a more negative body image. Indeed, empirical evidence is consistent with this hypothesis: AN patients report greater increases in negative affect after drinking water to the point of fullness (despite lower intake than healthy controls). This supports a direct link between GI distention and negative affect in EDs (T. A. Brown et al. 2022). Similar results have been reported for bulimia nervosa and binge ED, confirming a general link between fullness sensations and negative affect in EDs, regardless of weight (van Dyck et al. 2021). Negative affect is highly associated with body image (Pennesi and Wade 2016), providing a causal path between sensations of fullness and negative body image.

Because caloric intake is associated with sensations of fullness and satiety, it is easy to see how these sensory cues become associated with affect and ultimately body image. Boswell et al. (2015) suggest that individuals with higher interoceptive sensitivity may develop a restrictive ED if they view weight gain negatively, and “physical cues related to digestion (e.g., fullness) are experienced acutely as strong signals that have become associated with weight gain.” Through interoceptive conditioning, the sensation of fullness becomes perceived as a threat and drives avoidance behaviors, which are then negatively reinforced. Similarly, I suggest that sensations of emptiness or hunger are associated with thinness and thus safety and social reward, generating positive reinforcement. Dieting and weight changes would positively or negatively reinforce these associations through social rewards commonly conferred for weight loss and dieting efforts. Indeed, interoceptive sensitivity has been linked to greater interoceptive avoidance and body image disturbance (Zucker et al. 2013), and several studies (reviewed in Boswell et al. 2015) indicate that deficits in perceiving eating‐specific interoceptive signals predict dysregulated, emotional eating and mediate changes in eating symptom severity (A. J. Brown et al. 2010). Improvements in interoceptive awareness predict clinical outcomes in AN (Matsumoto et al. 2006), suggesting that awareness and acceptance of hunger and satiety cues may be important ED treatment targets (Herbert et al. 2012).

Further evidence for the link between GI sensations and body images comes from studies of GI pain and EDs. Early GI pain and discomfort may contribute to general aversive conditioning of GI signals; data suggest that early GI symptoms can increase the likelihood of later developing AN (Jacobi et al. 2004). It has been proposed that GI pain, as well as menstrual pain, increases preoccupation with gut sensations and disrupts their adaptive interpretation (Zucker and Bulik 2020). This could generalize to innocuous GI events such as fullness and satiety, providing a complementary variation of the process of amplification and negative interoceptive conditioning of GI distention, especially in females.

In chronic pain, nociceptive signals are interpreted as threatening, and patients often avoid certain movements and sensory experiences to avoid pain. Leading theories and psychological interventions for pain center on the reinterpretation of nociceptive signals as nonthreatening and movement as safe (Pinto et al. 2023; Ashar et al. 2022). Avoidance and dissociation are also common in both trauma and PTSD, where reduced insula processing of sensation has been observed; avoidance robs the patient of new experiences that could update predictive models to restore feelings of safety (Strigo and Simmons 2024). As in chronic pain, I propose that in EDs, relief from avoiding the perceived threat of fatness can reinforce avoidance behaviors, entrenching restrictive eating (Volders et al. 2015).

Individuals with EDs and affective disorders have been proposed to have challenges with self‐identity (Croce et al. 2024) that may lead to a greater focus on interoceptive input to evaluate self‐worth. It is unclear whether such challenges arise from internal or external factors, such as the difficulty of integrating stigmatized identities (e.g., LGBT adults experience a higher rate of EDs; Parker and Harriger 2020). Individuals with EDs may use starvation strategically to amplify hunger signals and avoid interoceptive uncertainty, strengthening their sense of self (Barca and Pezzulo 2020). Critically, however, this model does not explain why starvation is chosen over other intense sensory experiences. The current model proposes avoidance of GI fullness‐related sensations due to their specific link to fear of fatness and, consequently, negative affect and body image. Hunger signals are amplified to maximize interoceptive and affective processing associated with reward and safety, avoiding the “error signals” of fatness and aligning self‐identity with a hyperprior of the desirability and necessity of thinness.

### 2.4 GI Sensations Are Amplified via Central Sensitization

Central sensitization most commonly refers to the amplification of pain by central nervous system mechanisms (Harte et al. 2018). When a person is injured, nociceptors become sensitized to subsequent nociceptive stimuli and increase the excitability of central nociceptive processing (Cervero 1995). Attention and negative emotions further amplify pain processing in the brain (Apkarian et al. 2011), creating a positive feedback loop wherein pain, attention to pain, and fear of pain amplify pain processing and increase its likelihood of becoming chronic. As pain becomes chronic, body image is altered, including decreases in tactile acuity and interoceptive awareness of the body and disruptions of body image (Moseley 2008).

**FIGURE 1:**
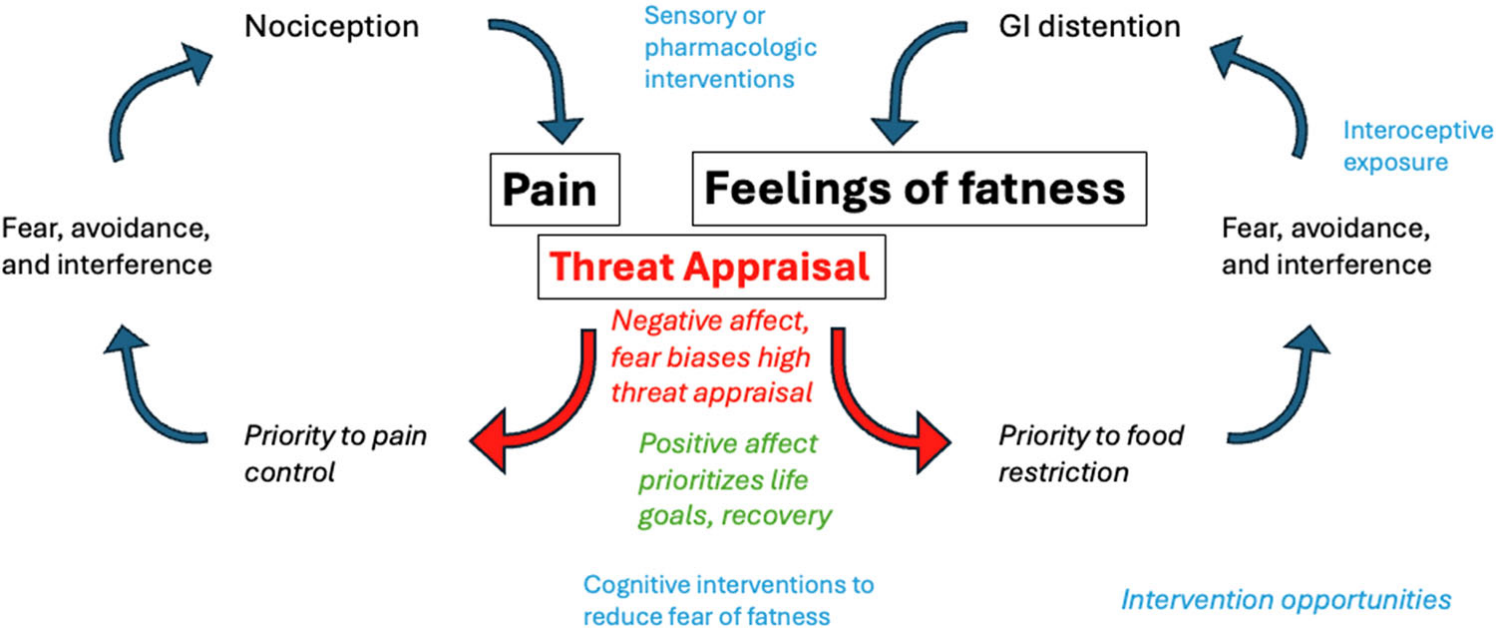
Parallel models of sensory amplification and threat detection for pain and fear of fatness. Elements of the model are drawn from the fear‐avoidance model of pain (Vlaeyen et al. 2016).

I propose a similar process by which gastric stretch sensations become amplified and interpreted as threatening. In EDs, attention and negative emotions are directed toward GI sensations of fullness and satiety, likely increasing their aversive central processing. I propose that the interpretation of these signals as threatening leads to central sensitization and increased salience of GI signals, increasing their intensity and leading to even greater aversion and avoidance. In the same way that central sensitization, negative affect, and avoidance create persistent pain, I propose that the same mechanisms apply to GI sensations in the context of fear of fatness, leading to persistent feelings of fatness and body image distortion (see Figure 1).

Central sensitization is plausible to consider in EDs given its application to visceral symptoms beyond pain. Central sensitization is believed to contribute to many chronic pain and hypersensitivity disorders, including irritable bowel syndrome, tension‐type headache, myofascial pain syndrome, PTSD, and more (Yunus 2008, and was recently proposed to contribute to overactive bladder (Reynolds et al. 2016). Indeed, central sensitization has been proposed as a risk factor for EDs (Sim et al. 2021), citing overlap in traits and behaviors between chronic pain and ED patients that may confer heightened sensitivity to internal and external environmental conditions, as well as interactions with stress, which are also implicated in the development of central sensitization (Adams and Turk 2015). Despite exhibiting increased acute pain tolerance, ED patients report high levels of persistent pain and extremely high levels of disorders of gut–brain interaction, consistent with a process of central amplification of GI pathways (X. Wang et al. 2014).

Indeed, individuals with EDs often exhibit higher interoceptive sensibility (IS) for visceral sensations, which are heightened after meal consumption (Jacquemot and Park 2020). This suggests heightened attention to these sensations. A third of AN patients even reported fullness when their stomach was completely empty (Bluemel et al. 2017), suggesting central amplification and distortion of fullness perception. Interoceptive accuracy (IAcc), in contrast, is reduced for hunger and satiety‐related cues in EDs, suggesting distortion in these signals. Since satiation is subjective, I suggest this is evidence of heightened sensitivity and central amplification of fullness sensations, rather than “faulty interoception.” Slowed gastric motility due to ED behaviors may further amplify sensations of fullness and distention (Schalla and Stengel 2019).

A validated and reliable measure of amplified gastric interoception (Van Dyck et al. 2016) has recently been demonstrated in the two‐step Water Load Test (WLT‐II), allowing a novel opportunity to study relationships between modulation of gastric interoception and body image. To complete the WLT‐II, participants ingest noncaloric water until perceived satiation (Step 1) and maximum fullness (Step 2) to determine gastric perception and discomfort thresholds (Van Dyck et al. 2016). The first step of the test has demonstrated lower thresholds for fullness sensations in individuals with AN (T. A. Brown et al. 2022), and WLT performance is correlated with IS, demonstrating convergent validity of the task (T. A. Brown et al. 2022). However, associations between WLT‐II and dieting or weight and shape concerns were not found in a nonclinical sample (Ahlich et al. 2023). Other promising tests include the administration of hydrogels and bioengineered vibrating capsules as probes for GI interoception (Greenway et al. 2019; Smith et al. 2021).

Altered gastric perception provides an empirical starting point from which to develop much‐needed mechanistic research on sensitization/amplification of GI‐related interoceptive signals associated with fullness and skin stretch. Model‐based experimental studies of interoception in EDs are needed to formally test for altered predictive processing (Khalsa et al. 2022) and psychological and neurobiological mechanisms by which GI interoception may become amplified. It would also be important to study whether peripheral sensitization occurs in the GI system in EDs (increased firing and lower threshold of sensory afferents in the GI system), as occurs to nociceptors in the skin and muscle in chronic pain. Peripheral inflammation could potentially be generated by antidromic processes secondary to central sensitization, as recently proposed in chronic musculoskeletal pain (Sikdar et al. 2023).

A contributor to amplification may involve neuroimmune mechanisms. Preclinically, glia and astrocytes can detect nerve injury in the spinal cord and are implicated in the consequent release of inflammatory mediators, after which touch becomes painful (Svensson and Brodin 2010; Gonçalves dos Santos et al. 2020). Neuroinflammation has recently been linked to amplification of central processing of pain signals in individuals with chronic pain (Loggia 2024). Recently, neuroinflammation has also been associated with mood alteration and mental fatigue (Brusaferri et al. 2022) and with persistent depressive and cognitive symptoms after SARS‐CoV‐2 infection (Braga et al. 2023). Research is needed to determine whether an altered interpretation of gut distention as threatening triggers an inflammatory response to GI signals, further amplifying and entrenching their aversive perception.

It is important to note that the framework proposed in the current paper does not apply to all EDs equally but to cases of restrictive eating. Individuals with bulimia nervosa and binge ED often report a lack of fullness, despite fear of fat. However, even if satiety signals are delayed or weakened, it remains the case that individuals with a fear of fatness are likely to interpret sensations of fullness—when they do arise—as threatening and indicative of fatness. Individuals may experience conflicting motivations to eat while simultaneously feeling the threat and negative interpretation of the sensation, which may then motivate compensatory behaviors. Indeed, individuals with bulimia nervosa exhibit worsened mood after binges, which returns to baseline after purging (Alpers and Tuschen‐Caffier 2001). In sum, even if fullness is delayed or not avoided, it may still be feared and interpreted as fatness and contribute unduly to negative body image and maintenance of ED behaviors.

## 3 Implications and Future Directions

Many AN patients fear losing self‐control (Froreich et al. 2016) and describe their bodies as dangerous and not trustworthy. (Arnaud et al. 2023) It has been argued that changes in interoception—specifically changes in *interoceptive sensibility—a*re merely by‐products of emotional changes, and that emotions play the central role in producing and maintaining behavioral symptoms of AN as identified in the DSM (Arnaud et al. 2023). In contrast, I propose that hyperpriors of prescriptive thinness and the social consequences of fatness drive the construction of emotions of disgust, danger, and mistrust from GI sensations of fullness. Thus, emotions are central, but equally so are the interoceptive sensations that underlie them and the cultural hyperpriors that link them. In addition, as sensations of fatness become amplified, larger prediction errors may be generated, leading to an upward spiral of emotional‐sensory amplification of GI signals, negative emotion, and fear of losing self‐control (a strong predictor of eating pathology; Froreich et al. 2016).

If fear‐based amplification of GI interoceptive channels is a key mechanism for feelings of fatness and food restriction, then we predict that desensitizing GI interoception should be highly beneficial for reducing distorted body image and restrictive eating. Interoceptive exposures are already supported as a transdiagnostic intervention strategy that can be integrated into cognitive‐behavioral therapy (CBT) for EDs (Boswell et al. 2015). Exposure strategies for food, body, and emotions have shown preliminary feasibility for residential ED treatment and support significant improvement in transdiagnostic outcomes for patients (Thompson‐Brenner et al. 2019). One novel interoceptive behavioral intervention attempted to decrease visceral sensitivity to the sensation of fullness/bloating and skills for tolerating emotions of distress and disgust associated with these sensations (Hildebrandt et al. 2021); Boswell et al. (2015) propose that both GI and emotion‐based somatic cues should be ED treatment targets, alongside attempts to increase tolerance and decrease fear, to disrupt the avoidance‐driven ED behaviors elicited by these sensations. The authors propose interoceptive interventions such as gulping water, wearing tight clothing, and jumping up and down. There is a great need for additional research on the effects of such visceral exposure and desensitization, and I propose that using water to decondition feelings of fullness from expectations and fears about calories and weight may be a novel clinical strategy that would be especially beneficial at deconditioning associations between fullness and weight gain.

In addition to GI‐focused exposure, I propose that psychoeducation about the neurobiological processes underlying amplified GI interoception might be a valuable clinical tool to provide patients with insight into the constructed nature of their feelings of fatness. Examples of sensitization to pain might provide a helpful analogy. CBT is a well‐supported therapy for EDs (Linardon et al. 2017) and can be used to challenge beliefs about the superiority of thin bodies as well as the link between sensations of fatness and body size. However, a recently validated novel therapy for nociplastic chronic pain (pain without any clear peripheral cause), *Pain Reprocessing Therapy*, has shown much higher effect sizes than typical CBT (Ashar et al. 2022). PRT validates the patient's experience of pain as a real sensation but guides them to reinterpret pain sensations as nonthreatening false‐alarm signals that—in the case of *chronic* pain—do not indicate injury or damage in the body. Applied to EDs, I propose that clinicians could validate the sensations of fatness the patient is experiencing, provide psychoeducation to explain their origin in amplified somatosensory pathways, and help the patient view these sensations as a misplaced alarm signal that is not indicative of either fatness or threat. In the treatment of chronic pain, *Pain Neuroscience Education* is one core component of PRT. Another core component of PRT is somatic tracking, which involves nonjudgmental attention to and description of pain sensations in the body—similar to the GI‐focused exposure and desensitization discussed above. Critically, the Pain Neuroscience Education occurs first, setting the framework in which patients can reinterpret their bodily sensations.

It is also worth emphasizing that despite progress, many ED providers and programs retain language and beliefs that are fatphobic, such as adhering to a prescriptive “normal” weight or BMI range for bodies, which will impede a patient's progress in reinterpreting the affective value assigned to fatness (Abel 2020). For patients to successfully reinterpret the affective value of bodily sensations, fatphobia must be eliminated. This is difficult but crucial work for ED treatment (Abel 2020; Stoll et al. 2022).

Furthermore, sensory strategies may be useful to *diminish* the salience of gastric distention and counter the effects of central sensitization. Indeed, in the treatment of pain, exposure therapies are used alongside sensory techniques and pain medications aimed at reducing the pain sensation itself. Deep pressure, for example, can inhibit visceral fullness sensations and decrease anxiety. In healthy adults, tactile stimulation reduces perception of gut stimuli, increasing tolerance to gut distention (Coffin et al. 1994). In addition, our research has shown that deep pressure is a form of affective touch that elicits sensations of pleasantness and decreases in anxiety (Case, Liljencrantz, McCall, et al. 2021) through a novel, non‐*Piezo2* sensory pathway (Case, Liljencrantz, Madian, et al. 2021). Deep pressure touch during massage induces greater calm than light touch (Field et al. 2010; Diego et al. 2004). Deep pressure has also recently been found to increase IAcc (Edwards et al. 2018), a function often impaired in AN (Pollatos et al. 2008). Of note, a case study observed remission from AN while wearing a pressured diving suit providing full‐body deep pressure (Grunwald 2005). Similarly, weighted blankets have decreased anxiety in inpatients with AN (Ohene et al. 2020). Research is also needed to test whether other sensory modalities can effectively diminish amplified GI interoception in ED patients. Heat, cold, vibration, and soothing affective touch therapies can all reduce the experience of chronic pain (Titler and Rakel 2001; McCaffery 1992) by gating pain signals in the spinal cord as well as engaging distraction and positive affective mechanisms in the brain (Villemure and Bushnell 2002). Furthermore, transcutaneous electrical nerve stimulation (TENS) and other noninvasive neuromodulation should be explored. These techniques can moderately reduce acute pain (Johnson et al. 2022), including visceral pain in GI disorders (Alam and Chen 2023).

In sum, I propose that fear and avoidance of fatness parallels the fear and avoidance that leads vulnerable individuals to treat pain signals as threatening, leading to central sensitization and chronification of pain. In EDs, this fear leads to an amplification of GI interoception that may be more central to ED behaviors than previously recognized. As sensations of fullness or distention are construed as signals of fatness, they become aversive and threatening, and the patient begins to avoid these sensations via further caloric restriction. While preliminary evidence supports each stage of this proposed model, more research is needed to confirm whether GI interoception is selectively amplified in EDs involving food restriction and whether GI‐focused interventions can diminish or reverse this sensitized pathway. Clinical research is needed to determine the feasibility and effectiveness of interventions focused on interoceptive desensitization, neuroscience education, and sensory interventions to diminish GI sensation.

## Author Contributions

**Laura Case**: conceptualization, writing – original draft, visualization, writing – review and editing, methodology, resources.

## Peer Review

The peer review history for this article is available at https://publons.com/publon/10.1002/brb3.70892.

## Data Availability

Data sharing is not applicable to this article, as no new data were created or analyzed in this study.
